# Factors Affecting Psychological Distress among People Living with HIV/AIDS at Selected Hospitals of North Shewa Zone, Amhara Region, Ethiopia

**DOI:** 10.1155/2019/8329483

**Published:** 2019-07-22

**Authors:** Elyas Admasu Basha, Behailu Tariku Derseh, Yohannes Gebre Egziabher Haile, Gedion Tafere

**Affiliations:** ^1^Debre Berhan University, Institute of Medicine and Health Science, Department of Nursing, Debre Berhan, Ethiopia; ^2^Debre Berhan University, Institute of Medicine and Health Science, Department of Public Health, Debre Berhan, Ethiopia; ^3^Debre Berhan University, Department of Psychology, P.O. Box 445, Debre Berhan, Ethiopia

## Abstract

**Background:**

The new advances for the treatment of HIV infection using Highly Active Antiretroviral Therapy (HAART) have dramatically improved disease prognosis. However, they are living longer with a chronic condition that increases the risk for psychiatric and psychosocial problems. Various studies have linked HIV/AIDS with a number of psychological problems, depression being the most common. Moreover, studies have found that chronically ill people are at increased risk of psychological problems. Thus, this study aimed at assessing the level of psychological distress and its associated factors among people living with HIV/AIDS in selected Hospitals of North Sowa Zone of Amhara region, Ethiopia, 2017.

**Method:**

Institution based cross-sectional study design with systematic random sampling method was used. Data was collected by structured interviewer-based Amharic version questionnaire. A total of 422 people living with HIV/AIDS were involved in the study from 1 to 30 May 2017. Data analysis was done with the help of a computer program (SPSS version 16.0). Binary logistic regression analysis was used for bivariate and multivariate analysis. The strength of the association was presented by odds ratio with a 95% confidence interval.

**Result:**

The prevalence of psychological distress was 7.8% (95% CI: 5.25%, 10.39%). Being female (AOR = 3.02; 95% CI: 1.16, 7.82), illiterates (AOR = 3.91; 95% CI: 1.31, 6.45), participants who currently use alcohol (AOR = 2.70; 95% CI: 1.23, 5.88), respondents whose CD4 count is less than 500 cells/*μ*l (AOR = 2.28; 95% CI: 1.02, 5.11), and participants who are considered stigmatized (AOR = 2.41; 95% CI: 1.11, 5.22) were positively associated with psychological distress.

**Conclusion:**

The prevalence of psychological distress was low as compared to other studies conducted in Ethiopia. This may affect the quality of life of people living with HIV/AIDS and their families. Being female, illiteracy, alcohol use, and having lower CD4 count and perceived stigma increased the odds of psychological distress. Thus, concerned stakeholders should collaborate on the integration of HIV/AIDs treatment and mental health services.

## 1. Introduction

The human immunodeficiency virus (HIV) epidemic was first identified in the 1980s [1, 2]. Since the start of the epidemic, around 78 million people have been infected with HIV and 39 million people have died with AIDS-related causes [2, 3]. Sub-Saharan Africa is the most affected region, with 25.6 million PLHIV, and accounts for two-thirds of the global total of new HIV infections [2–4]. According to the survey report from Ethiopian Public Health Institute in 2016, the national prevalence of HIV infection was 1.2% with the total estimation of 741,478 (291,414 males and 450,063 females) HIV infected people among the adult population [2, 3, 5].

Different HIV/AIDS prevention strategies and policies have been designed in order to combat the burden of the HIV/AIDS epidemic [2, 3]. In Ethiopia, there are prioritized interventions applied to fight against this chronic illness. HIV/AIDS chronic care is one of the prioritized strategies which include providing of ART for every HIV positive individual irrespective of their CD4 count and WHO HIV/AIDS staging in order to make these people live longer with improved quality of life. The first ART treatment was begun in 2003, and in 2005 free ART was started in Ethiopia. Currently, there are more than 1230 health facilities providing HIV care and treatment services. In these facilities, there are 367,000 adults and 23,400 children under the age of 15 taking ARV [2].

Opportunistic infections are the predominant causes of morbidity and mortality among HIV infected patients [2–4]. HIV affects the nervous system in 70-80% of infected patients [1, 2]. The result may be due to the direct effect of the virus, opportunistic infections, and/or malignancies [1–4]. Neurological manifestations of HIV can occur at any time from viral acquisition to the late stages of AIDs [1–3].

Up to 50 percent of persons with specific medical problems or stressors have been diagnosed with psychological problems [1]. Mental distress (or psychological distress) is a term used to describe a range of symptoms and experiences of a person's internal life that are commonly held to be troubling, confusing, or out of the ordinary.

A person in mental distress may exhibit some of the symptoms described in psychiatry, such as anxiety, confused emotions, hallucination, rage, depression, and others [6]. Several chronic medical illnesses like cancer, Parkinson's disease, Diabetes Mellitus, HIV/AIDS, and others are responsible for the development of psychological/mental distresses [7–10].

People living with HIV are at increased risk of developing a range of noncommunicable diseases (NCDs). With effective ART, people with HIV are living longer and experiencing NCDs associated with aging [2, 11, 12]. In general, around the world, mental health problems are more than twice as common among people living with HIV/AIDS compared to the general population [1, 13–17]. Since HIV/AIDS is a type of discriminating disease people with it are most frequently affected with lack of social support, poor self-esteem, stigma, and discrimination. This, in turn, predisposes them to psychological problems like depression and anxiety [11, 18–24]. In addition to the direct effect of HIV and the effect of social networking to cause psychological distress in PLWHA, the ART drug side effect might also be a predisposing factor for the development of these problems [1, 2, 25]. In Ethiopia, nearly half of the people living with HIV/AIDS were depressed or anxious [2]. The cooccurrence of other medical problems like tuberculosis outnumbers the occurrence of mental illness for individuals living with HIV that also magnifies the burden of the disease [26, 27]. Moreover, different studies indicated that the burden of HIV is dramatically becoming increased especially with the cooccurrence of different psychosocial problems. Therefore, the present study attempted to assess the level and associated factors of psychological distress in people living with HIV/AIDS on ART follow-up clinics.

## 2. Methods

### 2.1. Study Design, Setting, and Population

Institution-based cross-sectional study design was employed to determine the magnitude of and identify factors associated with psychological distress among HIV/AIDS patients selected from three public hospitals, Ethiopia. Out of 3123 PLWH following their ART and whose age was greater than or equal to 18 years were eligible for this study. However, study participants who were seriously ill had been excluded. These are HIV/AIDS patients who were Unable to give a response to the questions raised by the data collectors due to the severity of illness or comorbidities.

### 2.2. Sample Size and Sampling Procedure

The sample size was determined by using a single population proportion formula. The assumptions considered were (Z_*α*/2_) which is the standardized normal distribution value for the 95% confidence interval (1.96), the proportion of psychological distress (P) taken as 50% with the marginal error (d) of 5%. Moreover, considering 10% of the nonresponse rate, the total sample size calculated was 422. Systematic sampling technique was employed to recruit study participants. First, three hospitals were selected randomly by lottery method. Then, the sample size was allocated proportionally for each hospital (Debre Berhan Referral Hospital = 242, Enat district hospital = 152, and Ataye district hospital = 28). Finally, study participants were selected by using systematic random sampling in every sampling fraction (k=7) after selecting the first study participant by lottery method.

## 3. Study Variables

Dependent variable was prevalence of psychological distress.

Independent variables were the following: sociodemographic factors: age, sex, marital status, ethnicity, religion, occupation, educational status, and income; clinical characteristics: WHO HIV/AIDs stages, ART drug adherence, opportunistic infections, and duration of knowing their HIV status; and environmental factors: stigma and discrimination and substance use (Khat, alcohol, and tobacco).

## 4. Data Collection Instrument and Procedure

The questionnaire was first prepared in English and then translated into the national language, Amharic, and then again translated back to English to check for consistency and rephrasing difficult concepts. A structured, interviewer-based self-reported questionnaire (SRQ-20) [28] was used to measure psychological distress, which is validated in Ethiopia [29]. Each question has two options (Yes/No). Participants were given one point for each question if they answered “Yes” and given zero points if their response was “No.” Then, the total score was summed up and yielded a score ranging from 0 to 20. Finally, the score was recorded and a participant who scored greater than or equal to 11 was considered as having psychological distresses. Questions for assessing the sociodemographic, clinical history, and substance use status were designed from different pieces of literature. Factors related to perceived stigma due to HIV status were assessed by Stigma related experience scale (5-items) [10]. It measures stigma experiences of HIV/AIDS patients in a five-point Likert-type scale, that is, “never,” “rarely,” “sometimes,” “often,” and “always.” Items are then recoded into binary variables to reflect the presence or absence of each specific stigma experience in the way that “never,” “rarely,” and “sometimes” are recorded as “0” to reflect the absence of stigma and “often” and “always” are recorded as “1” to reflect the presence of stigma. Values are then summed across the five items for a scale score ranging from 0 to 5. So, the increase in the score of scale shows that respondents are experiencing more stigma and low score shows that respondents are experiencing lower levels of stigma. Data were collected by clinical nurses who work in the same health institution at the ART clinic. This helps us to keep the privacy and confidentiality of study participants.

## 5. Data Quality Control

Data quality was assured by designing proper data abstraction tools. The data collection instrument was pretested on 5% of the sample size out of the study area to avoid information contamination. Language clarity, appropriateness of data collection tools, estimating of the time required, and the necessary amendments were considered based on the pretest. Two-day training was given concerning the data collection tool and data collection process for both data collectors and supervisors. During the data collection time, close supervision and monitoring were carried out by supervisors and the principal investigator to ensure the quality of the data. Finally, all the collected data were checked by the supervisors and investigator for its completeness and consistency during the data management, storage, and analysis.

## 6. Data Processing and Analysis

Before analysis, data were cleaned, edited, and coded. Any errors identified at this time were corrected after reviewing the original data using questionnaire code numbers (001–422). Data was entered using Epi-Data version 3.1 and analyzed using SPSS version 16 statistical software. Descriptive statistic was used to explain the study participants in relation to study variables. Bivariate and multivariate logistic regression analysis was performed to identify associated factors with psychological distress. During binary logistic regression analyses, first, each variable was independently assessed to determine the crude odds ratio of each identified variable with psychological distress (P<0.2). After making an appropriate selection of variables having a strong association with the dependent variable, we moved to the second stage of analyses of multivariate logistic regression analyses of all variables found statistically significant during simple logistic regression (P<0.05). The strength of the association was presented by odds ratio with a 95% confidence interval.

## 7. Result

### 7.1. Sociodemographic Characteristics of the Study Participants

Out of the expected 422 respondents, all agreed to participate in the study, yielding a response rate of 100%. The mean age of the participants was 39.15 years (SD ± 10.44). Among the participants, 265 (62.8%) were females. One hundred seventy-nine (42.4%) of the participants were married. The majority, 378 (89.3%), of respondents were orthodox in their religion. Four hundred three (95.5%) of participants were Amhara in ethnicity. Regarding their occupational status, 127 (30.1%) of them were governmental workers. Three hundred (71.1%) of study participants were living alone (Table 1).

### 7.2. Clinical Characteristics, Substance Use, and Perceived Stigma of the Participants

Regarding the clinical characteristics, 363 (86%) of study participants know their HIV seropositive status before 12 months earlier the time of data collection. Half of the respondents (50.9%) have a CD4 count of less than 500 cells/*μ*L. Three hundred thirty-three (78.4%) of the participants were classified under the WHO clinical stage I and among the participants, 47 (11.1%) had history comorbidity called tuberculosis. One hundred twenty-nine (29.6%) of the respondents were currently using alcohol. One hundred fifty-nine (37%) of them have perceived stigma due to their illness (Table 2).

### 7.3. Prevalence of Psychological Distress

The prevalence of psychological distress among PLWHA is 7.8% (95% CI: 5.25%, 10.39%).

### 7.4. Factors Associated with Psychological Distress

Multivariate logistic regression analysis revealed that being a female, illiteracy, current alcohol usage, CD4 count less than 500 cells/*μ*l, and perceived stigma were statistically significant with psychological distress (p < 0.05). Being female participants (AOR = 3.02; 95% CI: 1.16, 7.82) were significantly associated with psychological distress. Illiterate participants were four times (AOR = 3.91; 95% CI: 1.31, 6.45) more likely to develop psychological distress than their counterparts. Similarly, respondents who were currently taking alcohol (AOR = 2.70; 95% CI: 1.23, 5.88), respondents whose CD4 count <500 cells/*μ*l (AOR = 2.28; 95% CI: 1.02, 5.11), and respondents who had perceived stigma (AOR = 2.41; 95% CI: 1.11, 5.22) were more likely to have psychological distress than their counterparts (Table 3).

## 8. Discussion

People with chronic illnesses are frequently at increased risk of developing physical, emotional, and psychological problems. HIV/AIDS is one of the chronic illnesses predisposing patients to these problems. This study identified gender (being female), illiteracy, current alcohol users, perceived stigma, and HIV infected individuals who had a CD4 count less than 500 cells/*μ*l as the determinants for psychological distress among PLWH receiving their ART at three selected hospitals of North Shewa Zone, Amhara regional state, Ethiopia, in 2017.

This study indicated that psychological distress among PLWH is 7.8%. This prevalence is in line with one cohort study done in Athena, Amsterdam (9.7%) [16]. In contrast, another research conducted in the Southern part of India revealed a slightly higher prevalence (12%). This might be due to the difference in study areas [17]. Similarly, psychological problems (depression and anxiety) among people living with HIV/AIDS are prevalent in most African countries [11, 24, 25] as compared with the present study. The discrepancy might be due to sociocultural, economic, study design, and environmental factor differences. The prevalence of psychological problems in two studies [26, 27] (one systematic review and one cross-sectional study) conducted in sub-Saharan Africa among PLWH is in line (9% and 8%, respectively) with the current study. Most of the studies conducted in Ethiopia showed that the prevalence of psychological problems is significantly higher than the prevalence of current study. For example, the prevalence of common mental disorders in one institution based cross-sectional study conducted in Ethiopia among TB/HIV coinfected patients is 63.7% [30]. This far discrepancy might be due to the presence of TB/HIV coexistence.

Being female is positively associated with psychological distress. Similarly, being female was positively associated with psychological distress in studies conducted in different parts of the world. For instance, studies conducted in South Africa (Cape Town) [18], Malaysia [20], Nigeria [11], Uganda [27], and Gondar, Ethiopia [31] showed that being female was statistically associated with psychological distress. The possible reason could be a biological (hormonal) difference with their counterparts. Female hormones like estrogen and progesterone imbalance are supposed to cause different psychological problems like anxiety and depression. The other assumption might be due to having less ability to bear traumatic life stressors like HIV/AIDS in females than males which makes them more vulnerable to develop adjustment problem with the illness.

On the top of sociodemographic factors, being illiterate is one of the factors associated with the development of psychological distress among the participants in this study. This association might be due to a lack of knowledge about the nature of the illness management options. As a result, they might be delayed to get appropriate investigation and management which gives a chance for the virus to replicate more rapidly and invade more host cells which lower their immunity. Due to this, they could be predisposed to develop different Opportunistic Infections (OIs) and their Central Nervous System (CNS) may be affected. This condition may also affect their cognition and emotion that would be manifested by anxiety and depression.

Moreover, the development of psychological distresses among study participants was associated with current alcohol use. Similarly, studies conducted in the Netherlands [16], Uganda [27], and Gondar, Ethiopia, [31] showed the association between alcohol use and distress. Many assumptions might be considered related to alcohol use and psychological distress. If people on any drug treatment including ART take alcohol, they would frequently forget to take their drugs at the right time and the right dose due to the effect of alcohol on parts of the brain which are responsible for memory, decision, and judgment. As a result, the drug could not function correctly and predisposes the patient to drug resistance and the virus continues to replicate and his/her immunity becomes lower. This, in turn, causes different OIs and psychological problems. Alcohol intake can also predispose patients to poor nutrition due to the physiological effect of suppression of the appetite center in the brain and poor absorption in the gastrointestinal (GI) system. Due to this reason, the immune system of the patient would be lowered and the psychological adaptation is less likely. The other effect of alcohol use during ART treatment regimen can be drug-alcohol interaction which makes the drugs ineffective and the opportunity to viral replication. This is also responsible for lowering of the body resistance to cause different psychological problems and OIs.

Another factor which was responsible for the development of psychological distress is CD4 cell count less than 500 cells/*μ*l. The same findings were reported from Cameron [24], Debark District Hospital Northwest, Ethiopia [32], and Dilla Hospital [33]. It is clear that when CD4 count becomes lesser in patients infected with HIV, the viral replication increases; this in turn decreases the body resistance of the individual which predisposes him/her to different OIs and malignancies. As a result, these OIs and malignancies might also affect the brain directly to cause different psychiatric problems.

Perceived stigma is another factor associated with psychological distress. Similarly, study conducted in India [17], South Africa (Cape Town) [18], northern Thailand and Zimbabwe [23], South Africa [25], Mekelle [34], and Alert Hospital [35] and a systematic review conducted in Ethiopia [36] and in Dilla Hospital [33] found that perceived stigma was positively associated with the development of psychological distress. Since HIV/AIDS is a stigmatizing and discriminating disease PLWHA could fear to have intimacy with others and they could be withdrawn from different social events. This makes them unable to have support in the daily challenges they could face. Moreover, the poor social interaction due to their perceived stigma might restrict them not to follow their chronic care regularly which leads to poor treatment adherence. Moreover, this could also cause poor psychosocial adjustment and create an opportunity for the virus to replicate continuously and the lowering of individuals' immunity which causes different OIs.

## 9. Strength and Limitation of the Study

The strength of this study is the fact that we enrolled an adequate number of study participants which helps us to assure the representativeness of the findings of the study to the entire cohort. Further, we have also attempted to measure multiple variables that have significance for psychological distress including perceived stigma. However, the findings of this study should be taken by considering the following methodological limitations. One of the shortcomings of this study could be recall bias but we tried to minimize it by including questions of recent memory. Moreover, during assessing alcohol intake behavior, we only measured whether they used or not. But, alcohol consumption without considering the content and volume of alcohol intake might have resulted in an underestimation of the strength of association with the outcome.

## 10. Conclusion

The prevalence of psychological distress was low as compared to other studies conducted in Ethiopia. Though the problem is relatively low, it may affect the quality of life of people living with HIV/AIDS and their families. Being female, illiteracy, alcohol use, having lower CD4 count, and perceived stigma increased the odds of psychological distress. Thus, concerned stakeholders should collaborate on integrating HIV/AIDs treatment service with mental health services. Behavioral change communication is mandatory to reduce risk behavior like alcohol use.

## Figures and Tables

**Table 1:** Sociodemographic characteristics of study participants at selected hospitals ART clinics, North Shewa Zone, Amhara regional state, Ethiopia, 2017 (n=422).

Variables	Frequency, N (%)
*Age group*	
18-24 years	22 (5.2)
25-34 years	110 (26.1)
35-44 years	274 (64.9)
>=45 years	16 (3.8%)

*Sex*	
Male	156 (37)
Female	266 (63)

*Ethnicity*	
Amhara	403 (95.5)
Others	19 (4.5)

*Religion*	
Orthodox	378 (89.6)
Muslim	17 (4)
Protestant	27 (6.4)

*Educational status*	
Illiterate	150 (35.5)
Literate	272 (64.5)

*Marital status*	
Married	179 (42.4)
Single	70 (16.6)
Divorced	72 (17.1)
Separated	38 (9)
Widowed	62 (14.7)

*Occupation*	
Governmental employed	128 (30.3)
Unemployed	63 (14.9)
Daily laborer	26 (6.2)
Housewife	85 (20.1)
Farmer	31 (7.3)
Merchant	61 (14.5)
Others	28 (6.6)

*Monthly income*	
<300 ETB	107 (25.4)
300-1000 ETB	126 (29.9)
1001-2500 ETB	83 (19.7)
>2500 ETB	106 (25.1)

Living condition	
Living with family	112 (26.5)
Living alone	300 (71.1)
Others	10 (2.4)

**Table 2:** Clinical, substance use and perceived stigma of the participants among PLWHA at three selected hospitals ART clinics, North Shewa Zone, Amhara regional state, Ethiopia, 2017 (n=422).

Variables	Frequency (n=422) (%)
*Last CD4 count*	
<500 cells/dl	215 (50.9)
=>500 cells/dl	207 (49.1)

*WHO stage*	
Stage I	331 (78.4)
Stage II	80 (19)
Stage III	11 (2.6)

*Opportunistic infection*	
Yes	54 (12.8)
No	368 (87.2)

*Types of OIs*	
TB	47 (87.04)
Herpes zoster	7 (12.96)

*Current tobacco use*	
Yes	8 (1.9)
No	414 (98.1)

*Current Khat use*	
Yes	14 (3.3)
No	408 (96.7)

*Current alcohol use*	
Yes	126 (29.9)
No	296 (70.1)

*Perceived stigma*	
Yes	159 (37.7)
No	263 (62.3)

**Table 3:** Factors associated with the psychological distress of the participants among PLWHA at three selected hospitals ART clinics, North Shewa Zone, Amhara regional state, Ethiopia, 2017 (n=422).

Variables	Psychological Distress		p-value	COR (95% CI)	AOR (95% CI)
	Yes	No			
*Sex*:					
Male	6	150	0.025	1	1
Female	27	239		2.86 (1.14, 7.14)	3.02 (1.16, 7.82)*∗*

*Education status*:					
Illiterate	21	129	0.001	3.53 (1.68, 7.39)	2.91 (1.31, 6.45)*∗*
Literate	12	260		1	1

*Marital status*					
Others	24	218	0.068	2.09 (0.95, 4.62)	-
Married	9	171		1	

*Alcohol use*					
No	15	281	0.002	1	1
Yes	18	208		3.12 (1.52, 6.67)	2.70 (1.23, 5.88)*∗*

*Perceived stigma*					
Yes	19	137	0.013	2.5(1.22. 5.26)	2.41 (1.11, 5.22)*∗*
No	14	252		1	1

CD4 count cells/*μ*l					
<500	23	187	0.02	2.48 (1.15, 5.36)	2.28 (1.02, 5.11)*∗*
>=500	10	202		1	1

COR = Crude Odds Ratio; AOR = Adjusted Odds Ration; CI = Confidence Interval; *∗* (asterisk) indicates the statistical association of predictors in multivariate logistic regression.

## Data Availability

The data used to support the findings of this study are included in this published article.

## Acronyms

AIDS: Acquired Immunodeficiency Syndrome
AOR: Adjusted Odds Ratio
ART: Antiretroviral Therapy
COR: Crude Odds Ratio
ETB: Ethiopian Birr
HAART: Highly Active Antiretroviral Therapy
HADS: Hospital Anxiety Depression Scale
HIV: Human Immunodeficiency Virus
NCDs: Noncommunicable Diseases
OIs: Opportunistic Infections
PLWH: People Living with Human Immunodeficiency Virus
SRQ-20: Self-report Questionnaire-20
TB: Tuberculosis
WHO: World Health Organization.

## References

[1] Sarkhel S. (2009). Kaplan and sadock's synopsis of psychiatry: behavioral sciences/clinical psychiatry, 10th edition. *Indian Journal of Psychiatry*.

[2] Ethiopian Federal Ministry of Health (2017). *National Comprehensive HIV Care and Treatment Training for Health Care Providers*.

[3] Ethiopian Federal Ministry of Health (2016). *National Comprehensive HIV Testing and Counseling Training Participant Manual*.

[4] Ethiopian Federal Ministry of Health (2014). *National Comprehensive HIV Care and Treatment Training for Health care Providers*.

[5] Central Statistical Agency [Ethiopia] and ICF International (2016). *Federal Democratic Republic of Ethiopia: Ethiopia Demographic and Health Survey 2016 Key Indicators Report*.

[6] National Institutes of Health (US) (2007). *Biological Sciences Curriculum Study. NIH Curriculum Supplement Series [Internet]*.

[7] National Institutes of Health (US) (2007). *Transforming the understandingand treatment of mental illnesses. NIMH mental health information [Internet]*.

[8] Paul A. A., Premraj F. C. Psychosocial problems and its impact faced by the HIV/AIDS infected patients. *IOSR Journal Of Humanities And Social Science*.

[9] Stetz K. M., Brown M. (2004). Physical and psychosocial health in family caregiving: a comparison of AIDS and cancer caregivers. *Public Health Nursing*.

[10] Rizalar S., Ozbas A., Akyolcu N., Gungor B. (2014). Effect of perceived social support on psychosocial adjustment of turkish patients with breast cancer. *Asian Pacific Journal of Cancer Prevention*.

[11] Obadeji A., Ogunlesi A. O., O Adebowale T. (2014). Prevalence and predictors of depression in people living with HIV/AIDS attending an outpatient clinic in Nigeria. *Iranian Journal of Psychiatry and Behavioral Sciences*.

[12] Hanass-Hancock J., Myezwa H., Carpenter B., Bellamy S. L. (2015). Disability and living with HIV: baseline from a cohort of people on long term ART in South Africa. *Plos One*.

[13] Gohil A. J., Parmar V. P. (2015). Family adjustment, social adjustment and depression in people with HIV positive diagnosis. *The International Journal of Indian Psychology*.

[14] Vanable P. A., Carey M. P., Blair D. C., Littlewood R. A. (2006). Impact of HIV-related stigma on health behaviors and psychological adjustment among HIV-positive men and women. *AIDS and Behavior*.

[15] Saki M., Mohammad Khan Kermanshahi S., Mohammadi E., Mohraz M. (2015). Perception of patients with HIV/AIDS from stigma and discrimination. *Iranian Red Crescent Medical Journal*.

[16] Schadé A., van Grootheest G., Smit J. H. (2013). HIV-infected mental health patients: characteristics and comparison with HIV-infected patients from the general population and non-infected mental health patients. *BMC Psychiatry*.

[17] Charles B., Jeyaseelan L., Pandian A. K., Sam A. E., Thenmozhi M., Jayaseelan V. (2012). Association between stigma, depression and quality of life of people living with HIV/AIDS (PLHA) in South India – a community based cross sectional study. *BMC Public Health*.

[18] Simbayi L. C., Kalichman S., Strebel A., Cloete A., Henda N., Mqeketo A. (2007). Internalized stigma, discrimination, and depression among men and women living with HIV/AIDS in Cape Town, South Africa. *Social Science & Medicine*.

[19] Varni S. E., Miller C. T., McCuin T., Solomon S. (2012). Disengagement and engagement coping with HIV/AIDS stigma and psychological well-being of people with HIV/AIDS. *Journal of Social and Clinical Psychology*.

[20] Barua A., Sharma Y., Basilio M. (2013). A psychosocial study on HIV/AIDS. *International Journal of Collaborative Research on Internal Medicine & Public Health*.

[21] Albrecht T. L., Adelman M. B. (1987). *Communicating Social Support: A Theoretical Perspective*.

[22] Forouzan A. S., Jorjoran S. Z., Sajjadi H. (2013). Social support network among people living with HIV/AIDS in Iran. *AIDS Research and Treatment*.

[23] Genberg B. L., Kawichai S., Chingono A. (2008). Assessing HIV/AIDS stigma and discrimination in developing countries. *AIDS and Behavior*.

[24] Ngum P. A., Fon P. N., Ngu R. C., Verla V. S., Luma H. N. (2017). Depression among HIV/AIDS patients on highly active antiretroviral therapy in the southwest regional hospitals of cameroon: a cross-sectional study. *Neurology and Therapy*.

[25] Pappin M., Wouters E., Booysen F. L. (2012). Anxiety and depression amongst patients enrolled in a public sector antiretroviral treatment programme in South Africa: a cross-sectional study. *BMC Public Health*.

[26] Bernard C., Dabis F., De Rekeneire N. (2017). Prevalence and factors associated with depression in people living with HIV in sub-Saharan Africa: A systematic review and meta-analysis. *Plos One*.

[27] Kinyanda E., Hoskins S., Nakku J., Nawaz S., Patel V. (2011). Prevalence and risk factors of major depressive disorder in HIV/AIDS as seen in semi-urban Entebbe district, Uganda. *BMC Psychiatry*.

[28] Beusenberg M., Orley J. H., World Health Organization - Division of Mental Health (1994). *A User's Guide to The Self-Reporting Questionnaire (‎SRQ /compiled by M. Beusenberg and J. Orley)*.

[29] Kortmann F. (1990). Psychiatric case finding in Ethiopia: shortcomings of the self reporting questionnaire. *Culture, Medicine and Psychiatry*.

[30] Deribew A., Tesfaye M., Hailmichael Y. (2010). Common mental disorders in TB/HIV co-infected patients in Ethiopia. *BMC Infectious Diseases*.

[31] Getinet Alemu W., Malefiya Y. D., Boru Bifftu B. (2016). Mental distress among patients admitted in gondar university hospital: a cross sectional institution based study. *Health Science Journal*.

[32] Bitew H., Andargie G., Tadesse A. (2016). Suicidal ideation, attempt, and determining factors among HIV/AIDS patients, Ethiopia. *Depression Research and Treatment*.

[33] Tesfaye S. H., Bune G. T. (2014). Generalized psychological distress among HIV-infected patients enrolled in antiretroviral treatment in Dilla University Hospital, Gedeo zone, Ethiopia. *Global Health Action*.

[34] Tesfay A., Gebremariam A., Gerbaba M., Abrha H. (2015). Gender differences in health related quality of life among people living with HIV on highly active antiretroviral therapy in mekelle town, Northern Ethiopia. *BioMed Research International*.

[35] Tesfaw G., Ayano G., Awoke T. (2016). Prevalence and correlates of depression and anxiety among patients with HIV on-follow up at Alert Hospital, Addis Ababa, Ethiopia. *BMC Psychiatry*.

[36] Amare T., Getinet W., Shumet S., Asrat B. (2018). Prevalence and associated factors of depression among PLHIV in Ethiopia: systematic review and meta-analysis, 2017. *AIDS Research and Treatment*.

